# Protective Effects of Lanosterol Synthase Up-Regulation in UV-B-Induced Oxidative Stress

**DOI:** 10.3389/fphar.2019.00947

**Published:** 2019-08-29

**Authors:** Hui Hua, Tianyao Yang, Liting Huang, Rentong Chen, Menglin Li, Zhenzhen Zou, Nan Wang, Dan Yang, Yang Liu

**Affiliations:** School of Public Health, China Medical University, Shenyang, China

**Keywords:** UV-B, lanosterol synthase, crystallin, oxidative stress, apoptosis

## Abstract

UV-B radiation may be an important risk factor in cataract etiology. After exposure to UV-B radiation, cells show imbalances in the repair of DNA damage, which induce changes in the levels of certain proteins, including alpha-crystallin, which is the most abundant protein in the lens and crucial for the maintenance of lens transparency. Lanosterol synthase (LSS), an essential rate-limiting enzyme in cholesterol biosynthesis, might play significant roles in oxidative stress and in the maintenance of lens transparency. However, the roles of LSS in UV-B-induced apoptosis are not well understood. Therefore, we irradiated female Sprague-Dawley rats with ultraviolet radiation to establish an animal model for exploring the variations in LSS expression during the early stages of UV-B exposure. In addition, we cultured human lens epithelial (HLE) cells that overexpress LSS and exposed them to UV-B radiation to explore the function of increased LSS expression in UV-B-induced apoptosis. The data demonstrated that UV-B exposure induced oxidative stress and apoptosis in rat lens epithelial cells and that irradiance exposure increased the level of lenticular damage. Additionally, UV-B exposure decreased the alpha-crystallin content and increased the expressions of Bax and cleaved caspase-3 compared with the control levels. After exposure to UV-B, the apoptosis-related index of HLE cells overexpressing LSS was lower than that of the control cells. Furthermore, ROS overproduction might activate the sirtuin 1 (Sirt1) pathway, which induced protein expressions of sterol regulatory element-binding transcription factor 2 (SREBF2), 3-hydroxy-3-methylglutaryl coenzyme A reductase (HMGCR), and LSS. However, the specific mechanism of the Sirt1 pathway needed to be further studied. In summary, UV-B exposure induced oxidative injury and resulted in crystallin denaturation and apoptosis in lens epithelial cells, and LSS might play a protective role during the early stages of this process and could be an important target in the cataract prevention.

## Introduction

Cataracts, which are predominantly related to the aging process (Zelentsova et al., 2017), remain the leading cause of legal blindness worldwide (Organization World Health; Bebbington, 2001)Epidemiological data showed that ultraviolet (UV) radiation was an important risk factor in cataract etiology (Delcourt et al., 2014) and could increase the global disease burden (Lucas et al., 2008). Sunshine is an important source of environmental UV. A previous study proposed a relationship between solar UV and risk for cataract development (Neale et al., 2003). Due to lens opacification, cataracts were directly caused by denatured crystallin (Moreau and King, 2012), and many studies have indicated that reactive oxygen species (ROS) was a crucial factor in cataracts by gradually inducing progressive lens opacity (Roberts, 2011; Osnes-Ringen et al., 2013; Xiang et al., 2018). Among the related features, pigmentation and apoptosis were prominent during this process (Linetsky et al., 2014; Galichanin, 2017), but the molecular mechanisms underlying UV-related cataract formation related to UV remain unclear.

Alpha-crystallin B (CRYAB) reportedly prevented H_2_O_2_-induced apoptosis in cardiac H9c2 cells (Xu et al., 2013), and similarly, alpha-crystallin A (CRYAA) blocked UV-A-induced apoptosis by activating the Akt survival pathway (Liu et al., 2004). Mutations in these crystallin proteins could cause cataracts (Mori et al., 2006). Lanosterol, a sterol found in the human lens, has been proposed to prevent the aggregation of mutated crystallin in humans (Zhao et al., 2015; Shen et al., 2018). Recent studies also demonstrated that lanosterol disrupted the aggregation of human γD-crystalline by binding to the hydrophobic dimerization interface (Kang et al., 2018), and lanosterol synthesis was likely correlated with oxidative stress processes (Hughes et al., 2007). Lanosterol synthase (2,3-oxidosqualene-lanosterol cyclase; LSS) catalyzes lanosterol formation, which was a key rate-limiting step in cholesterol biosynthesis (Huff and Telford, 2005; Nes, 2011).The incubation of Ganoderma lucidum with the pro-oxidant 1-chloro-2,4-dinitrobenzene could reduce LSS mRNA expression (You et al., 2012). These findings indicated that LSS might play significant roles in oxidative stress and the maintenance of lens transparency. However, the expression changes, and the roles of LSS in ROS-induced oxidative stress processes are not well known.

Under physiological conditions, cells maintained a dynamic balance between the oxidation and antioxidant defense systems, and UV radiation could disrupt this balance and thereby induced the production of oxidative stress in cells (Ayala et al., 2000). UV radiation was divided into three bands, and UV radiation B (UV-B) photons were five times more efficient in inducing mutations than UV radiation A (UV-A) photons (Dahle et al., 2005). A previous study reported that ROS, which was generated by UV-B, was involved in the transduction of at least some cellular signals (Heck et al., 2004). Oxidative stress induced autophagy (Lee et al., 2012) and apoptosis by damaging mtDNA and thereby inhibited the mitochondrial respiratory chain transition (Gredilla, 2010). In addition, previous studies have proposed that B cell lymphoma-2 (Bcl-2) family–related proteins modulated apoptosis and that cell fate depends on the ratio of Bcl-2-associated X protein (Bax) to Bcl-2 (Oltvai et al., 1993; Tzifi et al., 2012). Ricci *et al*. showed that the caspase pathway exerts feedback on permeabilized mitochondria during apoptosis under oxidative stress conditions (Ricci et al., 2003). ROS-induced damage was counteracted by the antioxidant systems in lenses, which included glutathione (GSH), superoxide dismutase (SOD), and other products related to redox reactions (Wojcik et al., 2010).

Therefore, we established a UV-B exposure model using female Sprague-Dawley (S-D) rats to evaluate the expression levels of LSS and other related proteins. The levels of oxidative stress and apoptosis in the lens after UV-B irradiation were investigated based on a series of related indicators. We attempted to explore the role of LSS during the early stages of UV-B-induced apoptosis in the human lens epithelial (HLE) cell line and to provide novel insights into the molecular basis of UV damage and early cataract prevention.

## Materials and Methods

### Chemicals

Pentobarbital sodium and tropicamide were obtained from Sigma (St. Louis, MP, USA), and phosphate-buffered saline (PBS), fetal bovine serum (FBS), and penicillin/streptomycin were purchased from HyClone (Los Angeles, CA, USA). Dulbecco’s modified Eagle medium-low glucose (DMEM-LG) and the Annexin V-FITC/PI Apoptosis Detection Kit were purchased from BD Biosciences (New York, NY, USA), and the SRA 01/04 cell line and lentiviruses were obtained from GeneChen (Shanghai, China). A cell proliferation assay kit (MTS) was obtained from Promega Biotech Co. (Beijing, China). Kits for the analysis of SOD, malondialdehyde (MDA), GSH, and glutathione peroxidase (GSH-Px) were purchased from Jiancheng Bioengineering Institute (Nanjing, China), and 2’7’-dichlorofluorescein diacetate (DCFH-DA) was obtained from Beyotime Biotechnology (Shanghai, China). TRIzol, PrimeScript^®^ RT Enzyme Mix I, SYBR Premix Ex TaqTM II Kit, and oligo (dT) primers were provided by TaKaRa Biotechnology Company (Dalian, China). Eyeball fixative was obtained from Wuhan Goodbio Technology (Wuhan, China), and kits for total protein extraction from cultured cells and tissues were obtained from Invent Biotechnologies, Inc. (MN, USA). Anti-Bax antibody, anti-Bcl-2 antibody, anti-cleaved caspase-3 antibody, and goat anti-rabbit IgG horseradish peroxidase (HRP) secondary antibody for immunohistochemistry (IHC) were purchased from Santa Cruz Biotechnology (CST) (CA, USA). Phenylmethylsulfonyl fluoride (PMSF) and bicinchoninic acid (BCA) reagent kits were obtained from Beyotime Biotechnology (Shanghai, China). The following immunoblotting antibodies were used: antibodies against Bax, cleaved caspase-3, and beta-actin were purchased from CST, and antibodies against Bcl-2, sterol regulatory element-binding factor 2 (SREBF2), LSS, 3-hydroxy-3-methylglutaryl-coenzyme A reductase (HMGCR), CRYAA&CRYAB, and Sirtuin1 (Sirt1) and goat anti-rabbit IgG H&L (HRP) secondary antibody were obtained from Abcam (Cambridge, UK). Resveratrol was obtained from Absin Bioscience Co. (Shanghai, China), and OneStep Western blocking solution and signal enhancer were obtained from APG Bio Ltd. (Sichuan, China).

### Animal Treatment and Lens Sample Collection

Six-week-old female S-D rats with initial body weights of 190–210g were obtained from the animal department of China Medical University (specific pathogen-free [SPF] grade; permit number: SCXK-2015-0001). All the animals were housed and treated in a normal indoor environment (temperature of 21–24°C and relative humidity of 30–40%) with food and water provided *ad libitum*. The animal room was maintained under a 12-h light/12-h dark cycle.

The rats were anesthetized through an overdose of 2% pentobarbital sodium (10 ml/kg) after the last UV-B exposure and were then subjected to cervical dislocation. The eyes were subsequently enucleated, and the lens of each eye was removed through a posterior scleral incision and cleaned with PBS. Most lens capsules were frozen and placed in liquid nitrogen before the experiments. The experiments were performed in strict accordance with the Guide for the Care and Use of Laboratory Animals of the National Institutes of Health. All surgeries were performed under anesthesia, and all efforts were made to minimize suffering.

### Cell Cultures, Transfection, and Addition of Sirt1 Activator

The HLE cell line SRA 01/04 was used in this study (the STR profile for this cell line is shown in **Supplemental Material 4**). The HLE cells used for the evaluation of ROS formation, apoptosis ratio, and oxidative and antioxidant contents were seeded in six-well cell culture plates, and the HLE cells used for western blotting were seeded in 100*20-mm cell culture dishes. The cells were cultured in DMEM-LG containing 10% FBS and 1% penicillin/streptomycin. The cells were incubated at 37°C and 5% CO_2_ and grown to 70% confluence for the experiments.

To assess the function of increased LSS expression in HLE cells exposed to UV-B, LSS was overexpressed in the experimental group. Lentiviruses containing the LSS overexpression sequence were purchased from GeneChen and transduced into HLE cells according to the manufacturer’s instructions, and these transduced cells were used as the experimental group. Some HLE cells were transfected with lentivirus containing a meaningless sequence and used as the control group (MOI = 50). The fluorescence-positive cells were observed by microscopy (fluorescence-positive cells obtained with different MOIs are shown in the **Supplemental Figure 1A** in **Supplemental Material 1**), and a western blot analysis of LSS was performed to assess the transfection efficiency (the protein bands obtained in the western blots are shown in the **Supplemental Figure 1B** in **Supplemental Material 1**).

To further explore whether the increase in LSS was related to a decrease in Sirt1 in HLE cells exposed to UV-B, we included cells pre-treated with resveratrol as an experimental group. A stock solution of resveratrol was obtained by dissolving resveratrol into absolute ethanol to a concentration of 100 mM. HLE cells were seeded in culture medium containing 50 μM resveratrol for 24 h before UV-B exposure. Twenty-four hours after the cells were exposed to UV-B, the expressions of Sirt1, SREBF2, HMGCR, and LSS proteins were evaluated by western blott.

### UV-B Exposure

#### UV-B Source

A medium-wave lamp with a central wavelength of 312 nm (Spectroline XX-15B, Spectronics Corporation, USA) composed of two unfiltered tubes and a Longlife filter. The typical peak UV intensity was 0.12 W m^−^² at 25 cm. The total UV radiation intensity reached the rat ocular, and the culture dish containing HLE cells was measured *via* a UV meter (UVX-31, UVP, USA).

#### Exposure of Rates to UV-B

Animals were divided into four groups: control and three UV-B irradiance groups. Rats in the UV-B groups were exposed to 1.5, 3.0, and 4.5 W m^−2^ UV-B irradiances for 15 min every other day. UV-B exposure was performed from 14:00 and lasted for three sessions, and the total doses to the rat eyes were 4,050, 8,100, and 12,150 J m^−2^. Animals were intraperitoneally injected with 2% pentobarbital sodium (3 ml/kg) for pre-anesthetization. To induce mydriasis, 10 mg/ ml tropicamide was instilled in both eyes and removed 3 min later before exposure in the UV-B irradiance groups, whereas the rats in the control group were only subjected to mydriasis.

#### Exposure of HLE Cells to UV-B

Prior to UV-B irradiation, the culture medium containing HLE cells in a petri dish was removed and replaced with 1 ml of PBS. To compare the differences between the HLE cells overexpressing LSS and the control cells, both the experimental and control cells were exposed to 1.5 W m^−2^ UV-B for 120 s (the exposure dose was 180 J m^−2^) based on the results from cell viabilities and morphological assessments (shown in **Supplemental Material Figures 1C, D** in **Supplemental Material 1**). After UV-B exposure, the PBS was discarded and replaced with culture medium, and the HLE cells were then cultured for 24 h in an incubator (Thermo, USA).

To explore the expression of cholesterol pathway–related protein after the addition of Sirt1 agonist, HLE cells were exposed to 1.5 W m^−2^ UV-B irradiance for 200 s (the exposure dose was 300 J m^−2^) based on previous studies (Chou et al., 2013). The exposure method was similar to previously described methods, and the HLE cells were cultured for 24 h in an incubator.

### Determination of MDA and Antioxidant Levels

Rat lenses were isolated from rat eyeballs and washed with PBS to prepare homogenate capsules. After exposure to UV-B for 24 h in a six-well cell culture plate, the HLE cells were collected using EDTA and DMEM-LG with 10% FBS.

The capsules and cells were reacted with thiobarbituric acid, and the level of MDA, a product of lipid peroxidation, was then determined *via* a colorimetric analysis (530 nm). To determine the antioxidant levels in lens epithelial cells after exposure to different levels of UV-B, the activities of SOD, GSH-Px, and GSH were measured using analytical kits. SOD activity was measured based on a WST-1 method according to the manufacturer’s instructions, and the absorbance was recorded at 450 nm. The rate for the catalytic reaction between GSH and H_2_O_2_ indicated the activity of GSH-Px. GSH activity was determined based on the principle that GSH could react with dithionitrobenzene (DTNB), and its specific activity was obtained through colorimetric determination at 550 nm. The absorbances at 412 and 405 nm were measured for the determination of GSH-Px and GSH activities, respectively.

### Apoptosis Assays

Fresh rat lens capsules were cut into small pieces, and then incubated with 0.25% trypsin-ethylenediaminetetraacetic acid (EDTA) for 15 min. The reaction was terminated by the addition of DMEM-LG containing 10% FBS. The mixture was then filtered through 200-µm mesh to obtain a single-cell suspension. The apoptotic rates of lens epithelial cells were measured by flow cytometry using an Annexin V-FITC/PI Apoptosis Detection Kit. In accordance with the manufacturer’s recommended protocol, the cells were washed twice with ice-cold PBS and resuspended in binding buffer to a density of 1 × 10^6^ cells/ml in the flow cytometry tube.

Based on a preliminary experiment (**Supplemental Material Figures 1B, C**), HLE cells were collected 24 h after UV-B exposure. The cells were digested with EDTA and DMEM-LG with 10% FBS in a culture dish, harvested after centrifugation, and re-suspended in binding buffer to a final concentration of 1×10^5^ cells/ml in the flow cytometry tube.

After, 10 μl of Annexin V-FITC and 5 μl of propidium iodide (PI) were added to the flow cytometry tube; the cells were incubated for 15 min in the dark at room temperature. Data for all the samples (each containing 1 × 10^4^ cells/ml) were collected by flow cytometry within 1 h and analyzed *via* FlowJo 7.6. In this study, the positive populations in the Q2 and Q3 quadrants (Annexin V+/PI+, Annexin V+/PI−) with respect to the entire cell population were determined to obtain the percentages of apoptotic cells.

### ROS Formation

Rat lens single-cell suspension was obtained as previously described. And after exposure to UV-B for 24 h, HLE cells were harvested using a method similar to that used for the apoptosis assays.

The intracellular formation of ROS was determined using an oxidation-sensitive fluorescent dye, 2’7’-dichlorofluorescein (DCFH). The cells were resuspended in DMEM-LG containing 10% FBS and DCFH-DA at a final concentration of 10 μM and incubated at 37°C for 30 min. The fluorescence at 488 nm (excitation)/525 nm (emission) was measured *via* flow cytometry and analyzed using FlowJo 7.6.

### Real-time PCR Analysis of Rat Lenses

Rat lenses were isolated from the eyeballs that had been previously washed with PBS, and capsules were removed. After homogenization, total RNA was extracted using the TRIzol reagent according to the manufacturer’s instructions and redissolved in RNase-free water. The absorbance of the RNA solution was determined using a NanoPhotometer (IMPIEN, Eppendorf, Germany) at 260 and 280 nm. The OD_260_/OD_280_ ratio was between 1.8 and 2.0, and cDNA was synthesized from total RNA by reverse transcription using PrimeScript^®^ RT Enzyme Mix I. Real-time quantitative PCR (RT-qPCR) was performed using the SYBR Premix Ex TaqTM II Kit and oligo (dT) primers using the ABI 7500 Real-Time PCR System (Applied Biosystems, USA). The final volume of the reaction mixture was 20 μl and contained 2 μl of template cDNA, 10 μl of SYBR, 0.8 μl of the PCR forward primer, 0.8 μl of the PCR reverse primer, 0.4 μl of the ROX reference dye II (50×), and 6 μl of dH_2_O. The real-time PCR cycle parameters included predenaturation for 30 s at 95°C followed by 40 cycles of 95°C for 5 s and 60°C for 34 s. The primer sequences used in this study are listed in **Table 1**. Gene expression was determined by the comparative CT method (^△△^CT) with respect to beta-actin gene expression.

**Table 1:** Primer sequences used in this study.

Name	Accession numbers	Sense primer	Antisense primer
Beta-actin	NM_031144.3	CATGTACGTTGCTATCCAGGC	CTCCTTAATGTCACGCACGAT
Bax	NM_017059.2	AAACTGGTGCTCAAGGCCCT	AGCAGCCGCTCACGGAG
Bcl-2	NM_016993.1	CCGGGAGAACAGGGTATGATAA	CCCACTCGTAGCCCCTCTG
Caspase-3	NM_012922.2	GAGCTTGGAACGCGAAGAAA	TCCACGGAGGTTTCGTTGTT
HMGCR	NM_013134.2	CCGGCAACAACAAGATCTGTG	ATGTACAGGATGGCGATGCA
LSS	NM_031049.1	TGGTTTCCTGCACATCCCTC	GTGGCGTAGCAGTAGCTCAT
Sirt1	XM_008772947.2	GAAAATGCTGGCCTAATAGACTTG	TGGTACAAACAAGTATTGATTACCG
SREBF2	NM_001033694.1	CTGCAGCCTCAAGTGCAAAG	CAGTGTGCCATTGGCTGTCT

Beta-actin was used as a housekeeping gene, and all data were normalized to beta-actin expression.

### Hematoxylin and Eosin (H&E) and IHC Staining of the Rat Lenses

Intact eyeballs were fixed with eyeball fixative for 48 h at room temperature, and the trimmed and fixed ocular tissues were dehydrated using a graded ethanol series (70%, 80%, 90%, 95%, and 100%) and vitrified with dimethylbenzene. The tissues were embedded into paraffin blocks, and the trimmed paraffin blocks were cut into 4-μm-thick sections. After deparaffinization, debenzolization using dimethylbenzene, and hydration through a graded ethanol series, the sections were stained with hematoxylin and eosin, and photographs were obtained under a light microscope. Anti-Bax antibody, anti-Bcl-2 antibody, anti-cleaved-caspase-3 antibody, and goat anti-rabbit IgG HRP secondary antibody were used for IHC staining.

### Quantification of HLE Cell Viability

The viabilities of HLE cells were measured by MTS assay. The cells were seeded and cultured in six-well cell culture plates, and the density of the cells prior to UV-B exposure was 2×10^5^/ml. After exposure to UV-B irradiance, each group of cells was cultured for 24, 48, and 72 h. The cells were then re-suspended to a final concentration of 1×10^5^ cells/ml and transferred to a 96-well assay plate. Subsequently, 20 μl of CellTiter 96^®^ AQueous One Solution Reagent was pipetted into each well, and the plate was incubated at 37°C for 1.5 h in an incubator. The absorbance values at 490 nm were measured using a microplate reader.

### Western Blotting

Rat lens homogenate capsules were prepared as previously described. Total protein was extracted using denaturing buffer (provided with the protein extraction kit) containing PMSF (denaturing buffer: PMSF = 100:1). HLE cells were collected 24 h after UV-B exposure by scraping from the culture dish using denaturing buffer containing PMSF. The protein concentrations were quantified using a BCA reagent kit.

Immunoblotting was performed using various primary antibodies, namely, Bcl-2 (1:800), Bax (1:800), cleaved caspase-3 (1:800), Sirt1 (1:800), beta-actin (1:5,000), SREBF2 (1:400), LSS (1:800), HMGCR (1:800), CRYAA&CRYAB (1:800), and goat anti-rabbit IgG H&L (HRP) secondary antibody (1:2,000). Enhanced chemiluminescence (ECL) substrate (Azure Biosystems c500, USA) was used for development of the protein bands obtained by western blotting. The band was semiquantitatively evaluated by densitometry using image analysis software (FluorChem v2.0, CA, USA). The relative expression of each protein was normalized to that of beta-actin, and similar data were obtained from at least two experiments.

### Statistical Analysis

The statistical analyses used in this study were performed using SPSS 22.0, and the data were found to exhibit normal distributions. The data from the animal experiments and the experiments in which Sirt1 agonist was added to cells were analyzed by one-way analysis of variance (ANOVA) followed by Dunnett’s t-test, and the data obtained from the experiments in which LSS was overexpressed in cells were analyzed by t-tests. Data were shown as the mean ± SD, and differences with either *P* < 0.05 (*) or *P* < 0.01 (**) were considered statistically significant.

## Results

### Effects of UV-B Exposure on Histological Analysis, ROS Formation, Crystallin, and Apoptosis Levels in Rat Lenses

**Figure 1:**
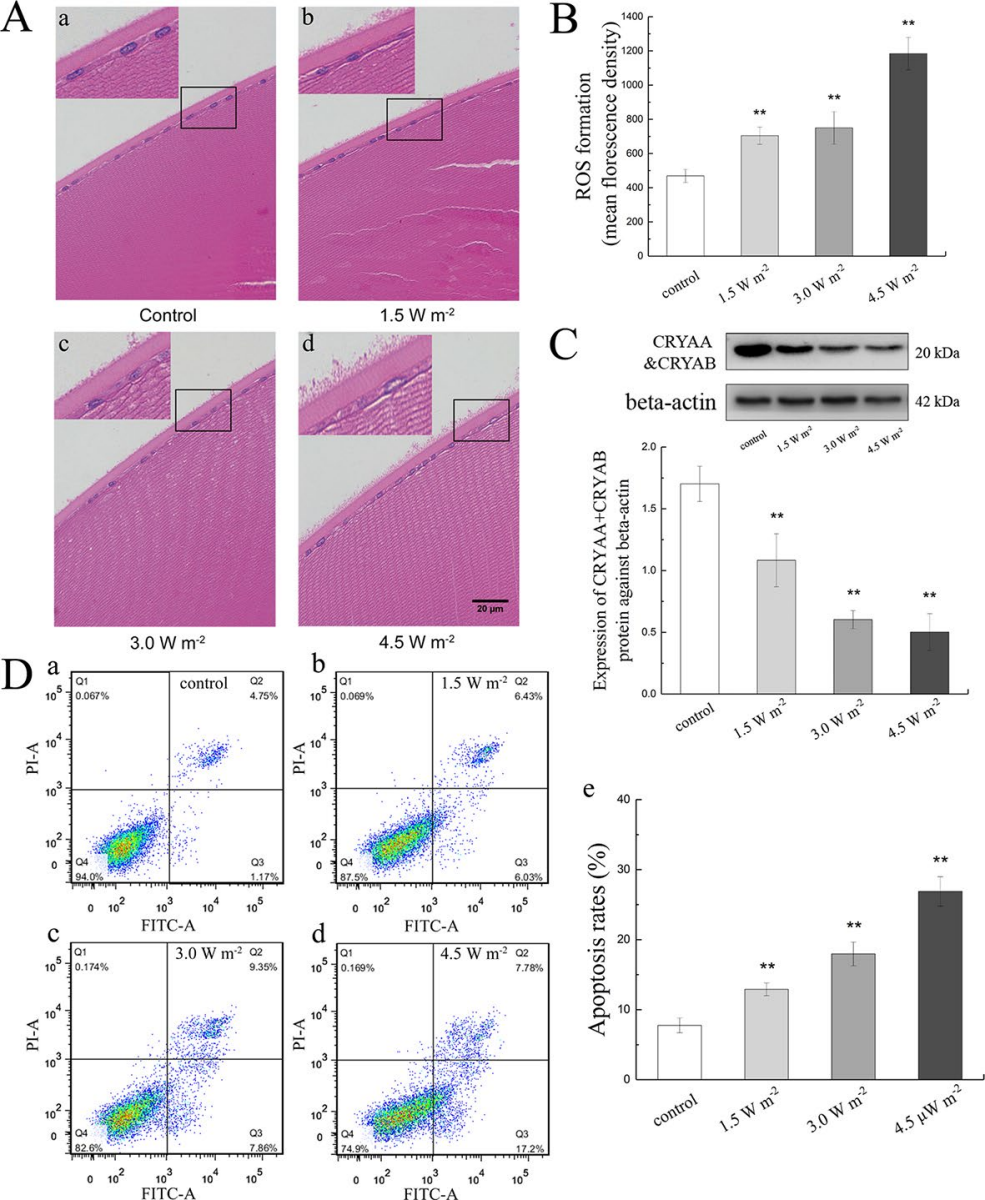
Effects of UV-B exposure on histological, ROS formation, crystallin, and apoptosis levels in rat lenses. It was shown in **Figure 1A** (400 ×) that lenses of rats treated with different UV-B intensities were stained with H&E. The effect of UV-B exposure on ROS formation in rat lens epithelial cells was shown in **Figure 1B**. The relative band densities of CRYAA and CRYAB proteins were quantified relative to the expression of beta-actin, and the results were shown in **Figure 1C** (n = 4). The apoptosis rates of dissociated lens epithelial cells analyzed by flow cytometry and the percentage of Annexin V-FITC+/PI− and Annexin V-FITC+/PI+ cells, which represented early and late apoptotic cells, respectively, were shown in **Figure 1D** (n = 6). Data were expressed as the mean ± SD. **P < 0.01 compared with the control group.

To explore the variation in the histology of rat lenses and the changes in ROS formation, apoptosis ratio, and the crystallin content in the rat lens epithelium after UV-B exposure, we constructed an animal model by irradiating female S-D rats with UV-B radiation. The rat lenses appeared to exhibit architectural lens damage, and the degree of damage increased with increases in the UV-B doses to which the rat eyes were exposed. The lens cells in the control group were closely and regularly spaced and showed a linear arrangement. UV-B treatment induced proliferation of the lens epithelial cells, and the analysis of the cells exposed to UV-B revealed irregular nuclei and intercellular spaces with many vesicles and water splits. The degree of architectural damage increased with increases in the exposure dose (**Figure 1A**). In addition, the ROS levels of the rats exposed to UV-B was increased significantly compared with those of the control group, and increases in the UV-B doses (1.5, 3.0, and 4.5 W m^−2^) increased the level of ROS formation (**Figure 1B**, *P* < 0.01). Western blot analysis clearly showed that the CRYAA and CRYAB protein expression levels decreased in a dose-dependent manner (**Figure 1C**, *P* < 0.01). Under these conditions, we detected the apoptotic levels of rat lens epithelial cells after UV-B exposure. The percentages of apoptotic (Q2 and Q3 quadrants in **Figure 1D**, **a**–**d**) increased in a UV-B dose-dependent manner, and the apoptotic rates of the rat lenses exposed to UV-B were markedly increased compared with those of the control group (*P* < 0.01, **Figure 1D**, **e**).

### Effects of UV-B Exposure on MDA, SOD, GSH, and GSH-Px Activities in Rat Lenses

**Figure 2:**
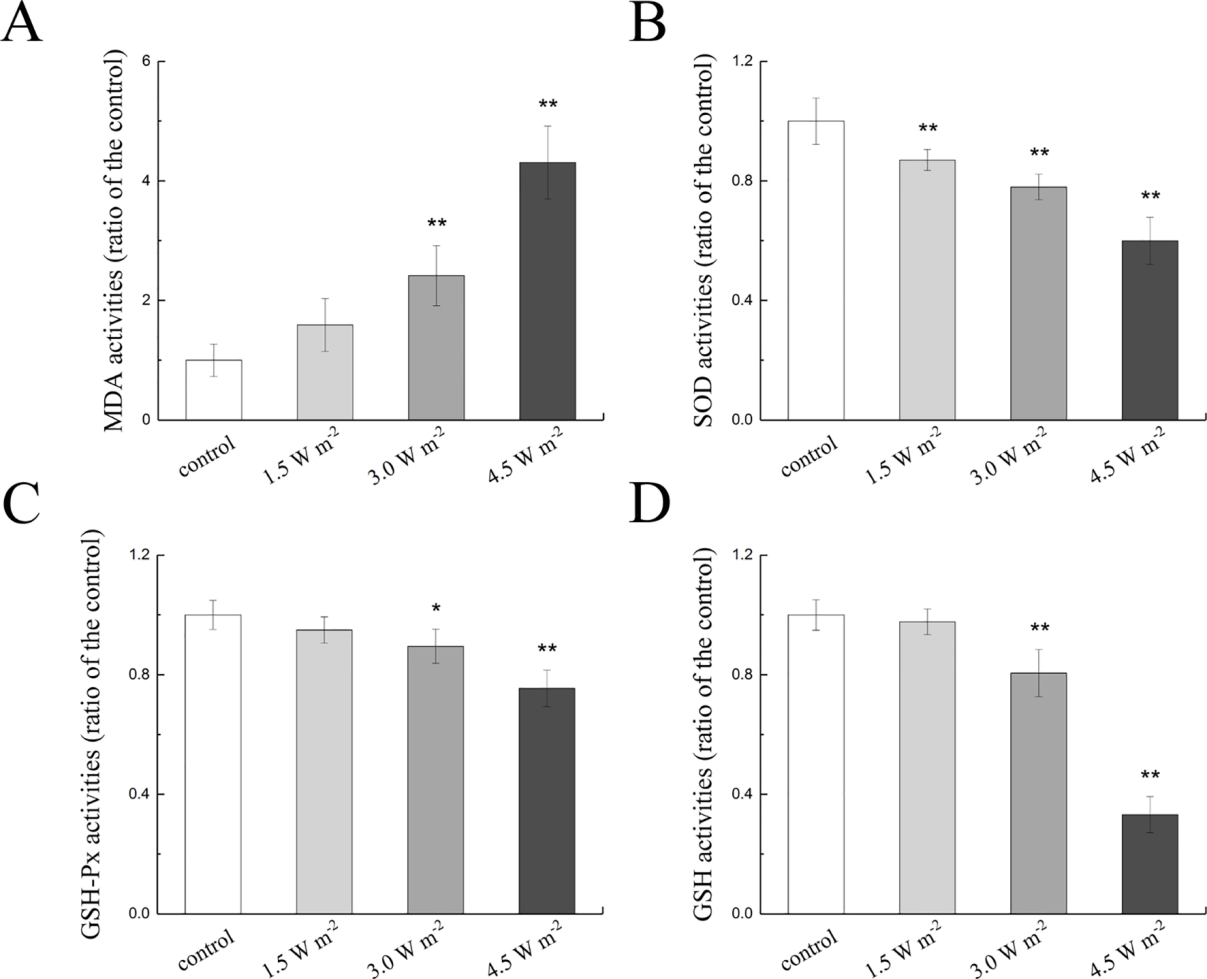
Effects of UV-B exposure on MDA, SOD, GSH-Px, and GSH activities in rat lenses (shown in **A** to **D**). The results were expressed as the ratio of the activity in the experimental group to that of the control group. Data were expressed as the mean ± SD. *P < 0.05, **P < 0.01 compared with the control group.

The measured MDA, SOD, GSH-Px, and GSH activities in the rat lens epithelium are shown in **Figure 2**. The rat eyes were exposed to 1.5, 3.0, and 4.5 W m^−2^ UV-B and exhibited dose-dependent increases in MDA activity to values that were 1.59-fold, 2.41-fold (*P* < 0.01), and 4.31-fold (*P* < 0.01) higher than the control levels, respectively (**Figure 2A**). In addition, the SOD activities exhibited significant concentration-dependent decreases after exposure to UV-B compared with those found in the control group (*P* < 0.01, **Figure 2B**). In contrast, 3.0 and 4.5 W m^−2^ UV-B exposure significantly decreased the activity of GSH-Px by almost 89.52% (*P* < 0.05) and 75.47% (*P* < 0.01) of the control GSH-Px activity (**Figure 2C**), respectively, and significantly decreased the activity of GSH by 80.57 and 33.16% of the control GSH activity (*P* < 0.01, **Figure 2D**), respectively.

### Effects of UV-B Exposure on Apoptosis-Related Protein Expression in Rat Lenses

**Figure 3:**
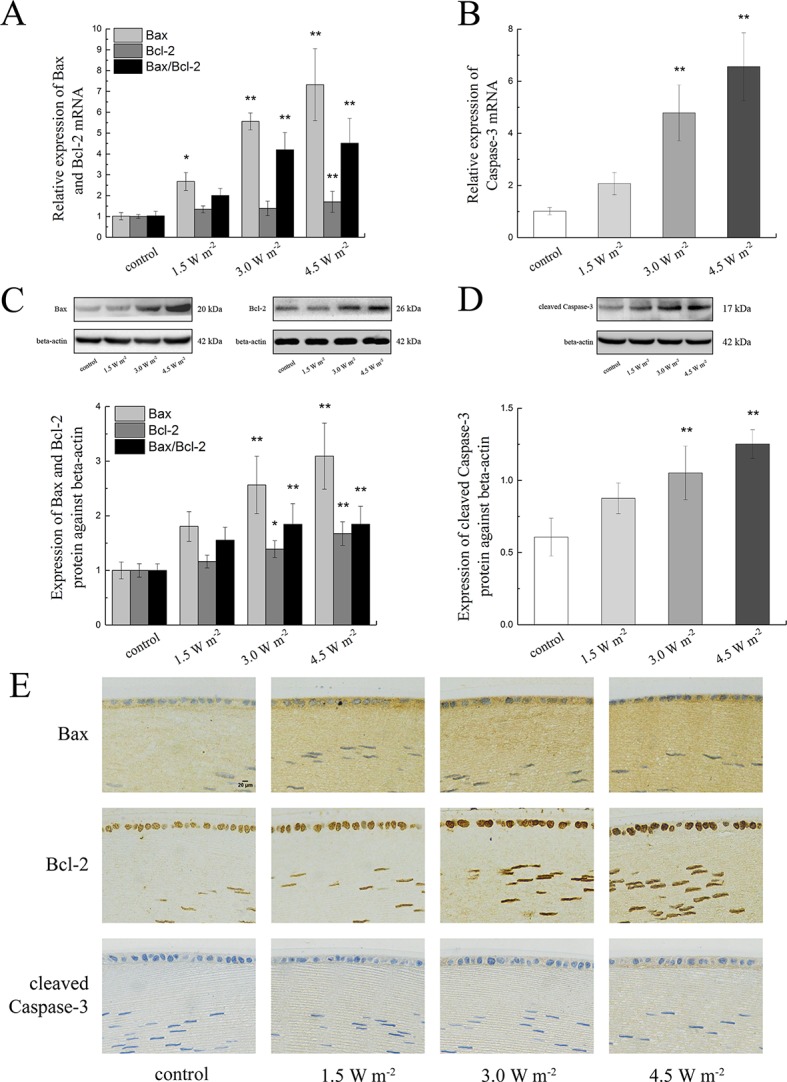
Alterations in Bax, Bcl-2, and caspase-3 mRNA and protein expression in rat lenses. The relative expression levels of *Bax, Bcl-2, and caspase-3* mRNA normalized to that of the housekeeping gene (**beta-actin**) were shown in **(A**, **B)** (n = 6). The relative band densities of Bax, Bcl-2, and cleaved caspase-3 proteins were quantified relative to the expression of beta-actin, and the results were shown in **(C**, **D)** (n = 4). An immunohistochemical analysis of a single protein after exposure to four UV-B doses was shown in **(E)** (400 ×). The experiment was performed twice with similar results. Data were expressed as the mean ± SD. *P < 0.05, **P < 0.01 compared with the control group.

To explore the changes in the expression of apoptosis-related protein in the rat lens epithelium after UV-B exposure, the Bax, Bcl-2, and caspase-3 expression levels after the UV-B exposure were quantified. The gene expression levels of *Bax*, *Bcl-2,* and *caspase-3* were measured by semiquantitative real-time PCR analysis, and the measured mRNA expression levels after exposure to UV-B are shown in **Figures 3A, B**. As shown, compared with that of the control group, the ratio of *Bax* to *Bcl-2* mRNA expression increased in a concentration-dependent manner; specifically, levels that were almost 4.14-fold and 4.45-fold higher than the control level were observed after exposure to 3.0 and 4.5 W m^−2^ (*P* < 0.01), respectively.

Consistent with the results from the gene expression analysis, the Bax and Bcl-2 protein expression levels were markedly increased in a dose-dependent manner after UV-B exposure compared with the control levels, and similarly, the ratio of Bax to Bcl-2 protein expression also showed a concentration-dependent increased compared with the control ratio (**Figure 3C**). The western blot results illustrated in **Figure 3D** showed that UV-B exposure increased the expression of cleaved caspase-3 in a dose-dependent manner. Immunohistochemical staining of the rat crystalline lens sections showed that increases in the UV-B exposure dose increased the protein expression levels of Bax, Bcl-2, and cleaved caspase-3. Bax immunopositivity was observed in the cytoplasm of the lens epithelial cells and fibers, and conversely, Bcl-2-immunopositive was detected in the nuclei. Cleaved caspase-3 protein mainly showed immunopositivity in the cytoplasm of the lens epithelial cells and fibers. Moreover, the mean optical densities of Bax, Bcl-2, and cleaved caspase-3 increased after UV-B exposure (**Figure 3E**).

### Effects of UV-B Exposure on Sirt1, SREBF2, HMGCR, and LSS Expression Levels in Rat Lenses

**Figure 4:**
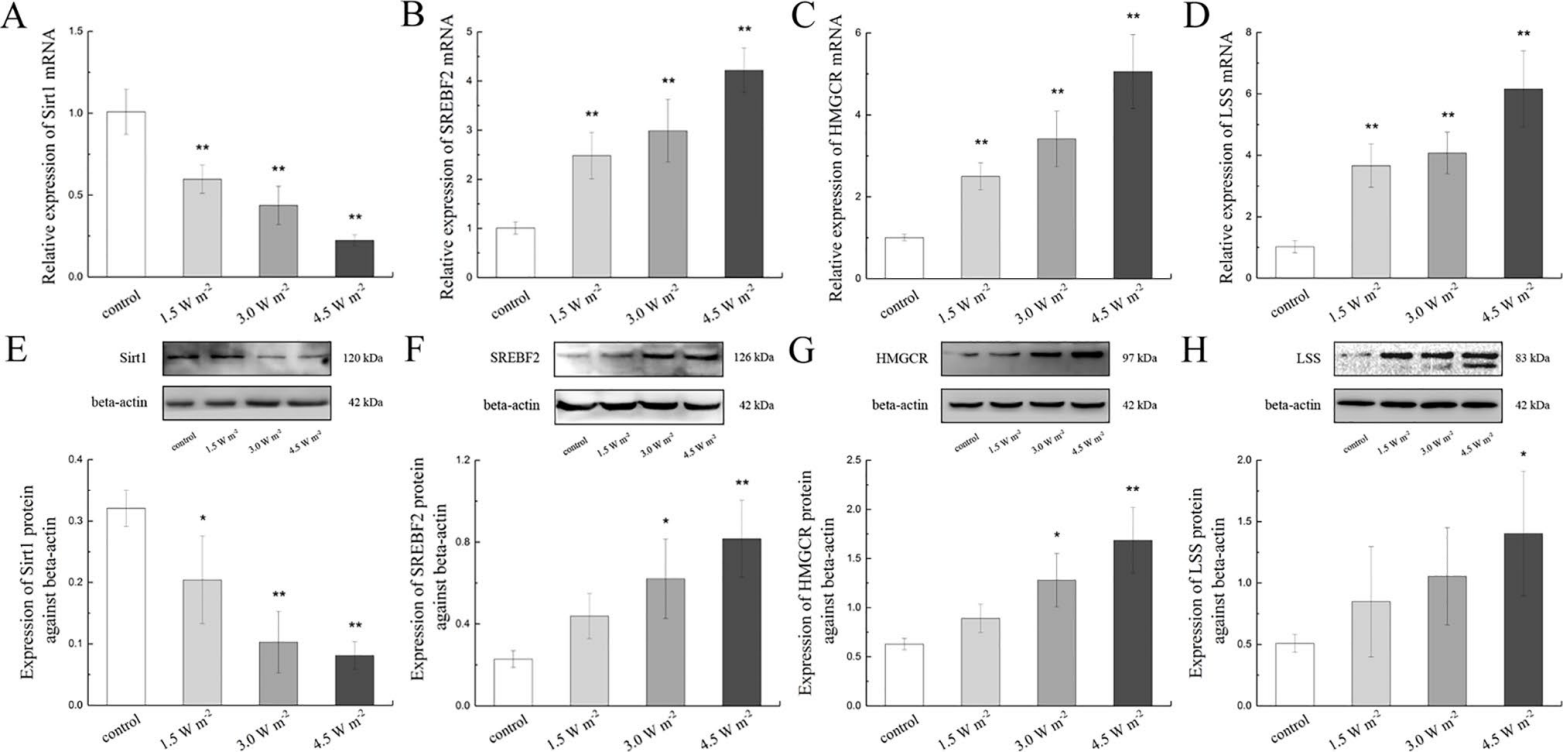
Sirt1, SREBF2, HMGCR, and LSS mRNA and protein expression alterations in the lens after UV-B exposure. The relative expression levels of Sirt1, *SREBF2*, *HMGCR*, and *LSS* mRNA normalized to the housekeeping gene (*beta-actin*) were shown in **(A)** to **(D)** (n = 6). The relative band densities of Sirt1, SREBF2, HMGCR, and LSS proteins were quantified relative to the expression of beta-actin, and the results were shown in **(E)** to **(H)** (n = 4). The experiment was performed twice with similar results. Data were expressed as the mean ± SD. *P < 0.05, **P < 0.01 compared with the control group.

To explore the changes in the expressions of LSS and related proteins in the rat lens epithelium after UV-B exposure, the *Sirt1*, *SREBF2*, *HMGCR,* and *LSS* gene expression levels were quantified after UV-B exposure. As shown in **Figure 4A**, UV-B exposure might down-regulate *Sirt1* gene expression (*P* < 0.01). Conversely, UV-B exposure significantly up-regulated the *SREBF2*, *HMGCR,* and *LSS* mRNA expression levels in a dose-dependent manner compared with those of the control group (*P* < 0.01, **Figures 4B–D**).

A western blot analysis was also performed to determine Sirt1, SREBF2, HMGCR, and LSS protein expressions. Consistent with the gene expression results, the Sirt1 protein expression levels decreased in a dose-dependent manner compared with those of the control, and this change was particularly observed after exposure to 4.5 W m^−2^ UV-B (*P* < 0.01, **Figure 4E**). Consistent with their corresponding gene expression, the protein expression levels of SREBF2, HMGCR, and LSS increased significantly in a UV-B dose (1.5, 3.0, and 4.5 W m^−2^)-dependent increased compared with the control levels (**Figures 4F–H**). These data indicate that the expression of LSS in the rat lens was increased during the early stages after UV-B exposure, and that this process might be related to the Sirt1 pathway.

### Effects of UV-B Exposure on Cell Morphology, Cell Viability, ROS Formation, Apoptosis Levels, Crystallin Alterations, and LSS Protein Expressions in HLE Cells

**Figure 5:**
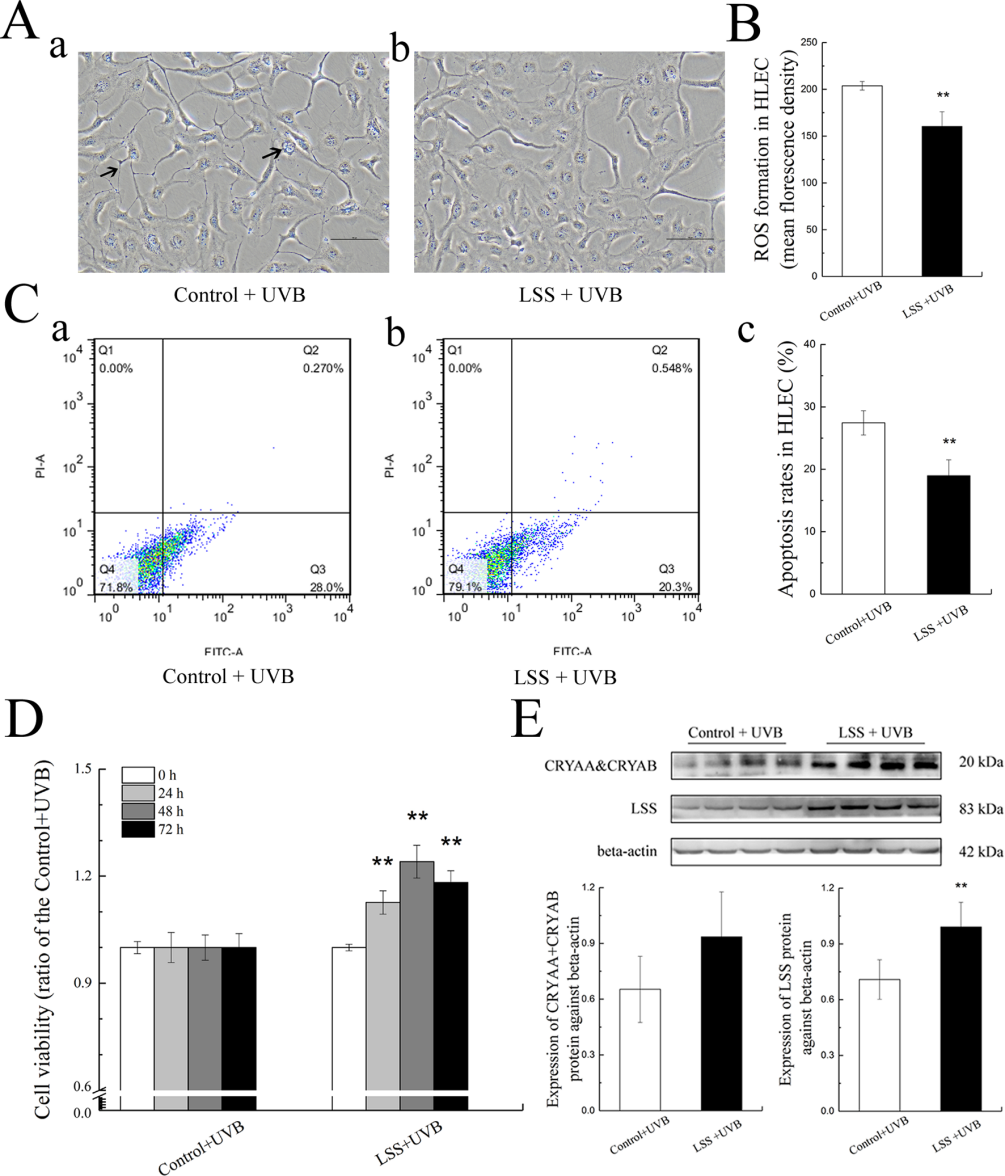
Effects of UV-B exposure on cell morphology, cell viabilities, ROS formation, apoptosis levels, crystallin alterations, and LSS protein expression in HLE cells. Control HLE cells and LSS-overexpressing HLE cells were exposed to UV-B radiation. The morphological changes in HLE cells after UV-B exposure were shown in **(A)**. The effect of UV-B exposure on ROS formation in HLE cells was shown in **(B)**. The apoptotic rates of HLE cells and percentages of Annexin V-FITC+/PI− and Annexin V-FITC+/PI+ cells, which correspond early and late apoptotic cells, respectively, were shown in **(C)** (n = 6). The cell viabilities at the time points of 24, 48, and 72 h were shown in **(D)** (n = 6). The relative band densities of LSS and crystalline protein expression were quantified relative to the expression of beta-actin, and the results were shown in **(E)** (n = 4). The experiment was performed twice with similar results. Data were expressed as the mean ± SD. **P < 0.01 compared with the control+UV-B group.

To explore the role of increased LSS protein expression, we overexpressed LSS in HLE cells and exposed these experimental HLE cells as well as control HLE cells to 1.5 W m^−2^ UV-B and investigated the subsequent alterations in cell morphology, cell viability, ROS formation, apoptosis level, crystallin, and LSS protein expression. After exposure to UV-B radiation, fewer HLE cells, increased cell vacuolar degeneration, and thinner intercellular adhesion were observed in the control group, compared with the LSS overexpression group (**Figure 5A**, arrows indicate thinner cell junctions and intracellular vacuoles). Under the same UV-B exposure conditions, the ROS levels in the HLE cells were significantly decreased in the overexpression group to 78.65% of the control levels (*P* < 0.01, **Figure 5B**). Consistent with the ROS level, the apoptotic percentage of the overexpression group was markedly decreased compared with that of the control group (*P* < 0.01, **Figure 5C**). Subsequently, cell viability was decreased in a time-dependent manner by UV-B exposure compared with that of the control group. Specifically, after exposure to UV-B radiation, the LSS overexpression group exhibited increased cell viability compared with the control group at the time points of 24, 48, and 72 h, markedly (**Figure 5D**). A western blot analysis revealed that exposure to UV-B radiation resulted in higher crystallin and LSS protein expression levels in HLE cells. As shown in **Figure 5E**, after exposure to UV-B radiation, higher CRYAA and CRYAB soluble protein contents were detected in the LSS-overexpressing HLE cells compared with the control cells. Furthermore, the LSS protein expression level in the LSS overexpression group was significantly increased by almost 1.40-fold compared with that of the control group (*P* < 0.01).

### Effect of UV-B Exposure on MDA, SOD, GSH-Px, GSH Activities, and Apoptosis-Related Protein Expression Alterations in HLE Cells

**Figure 6:**
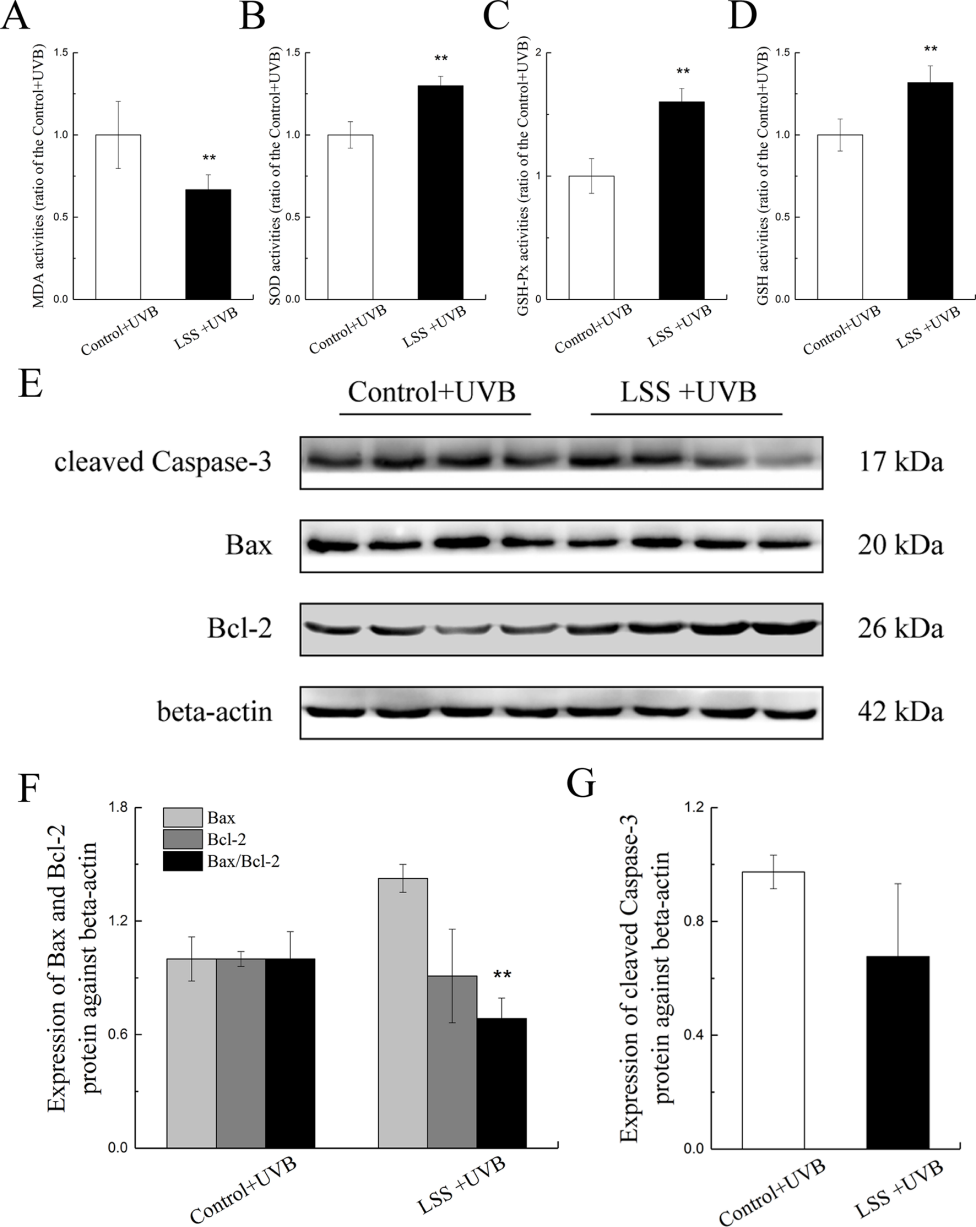
Effects of UV-B exposure on MDA, SOD, GSH-Px, and GSH activities and alterations in Bax, Bcl-2, and cleaved caspase-3 protein expressions in HLE cells. Control HLE cells and LSS-overexpression HLE cells were exposed to UV-B radiation. The effect of UV-B exposure on MDA, SOD, GSH, and GSH-Px activities were shown in **(A)** to **(D)**. The results were expressed as the ratios to the control levels (n = 6). The relative band densities of Bax, Bcl-2, and cleaved caspase-3 protein expressions were quantified relative to the expression of beta-actin, and the results were shown in **(E)**, **(F)**, and **(G)** (n = 4). The experiment was performed twice with similar results. Data were expressed as the mean ± SD. **P < 0.01 compared with the control+UV-B group.

**Figures 6A D** show the MDA, SOD, GSH-Px, and GSH activities in HLE cells after UV-B exposure. Compared with the control group, the MDA content in the LSS overexpression group was decreased to 66.73% of that found in the control group, and conversely, the SOD activity showed a 1.35-fold increase (*P* < 0.01, **Figure 6A, B**). Moreover, after exposure to UV-B radiation, in the LSS overexpression group, the activity of GSH-Px and the content of GSH were increased to 1.60-fold and almost 1.32-fold compared with the control levels, respectively (*P* < 0.01). The alterations in apoptosis-related protein expression in HLE cells after UV-B exposure were quantified (**Figures 6E–G**). Relative to that in the control group, Bax protein expression was decreased by almost 0.91-fold in the LSS overexpression. In contrast, Bcl-2 protein expression was increased, but the difference was not significant. Furthermore, the Bax-to-Bcl-2 protein expression ratio in the LSS overexpression group was significantly decreased by 0.68-fold compared with that of the control group (*P* < 0.01, **Figure 6F**). The expression of cleaved caspase-3 was decreased in the LSS overexpression group to 69.44% of that in the control group (**Figure 6G**).

### Alterations in Cholesterol Synthesis Pathway–Related Protein Expression After UV-B Exposure and Resveratrol Treatment in HLE Cells

**Figure 7:**
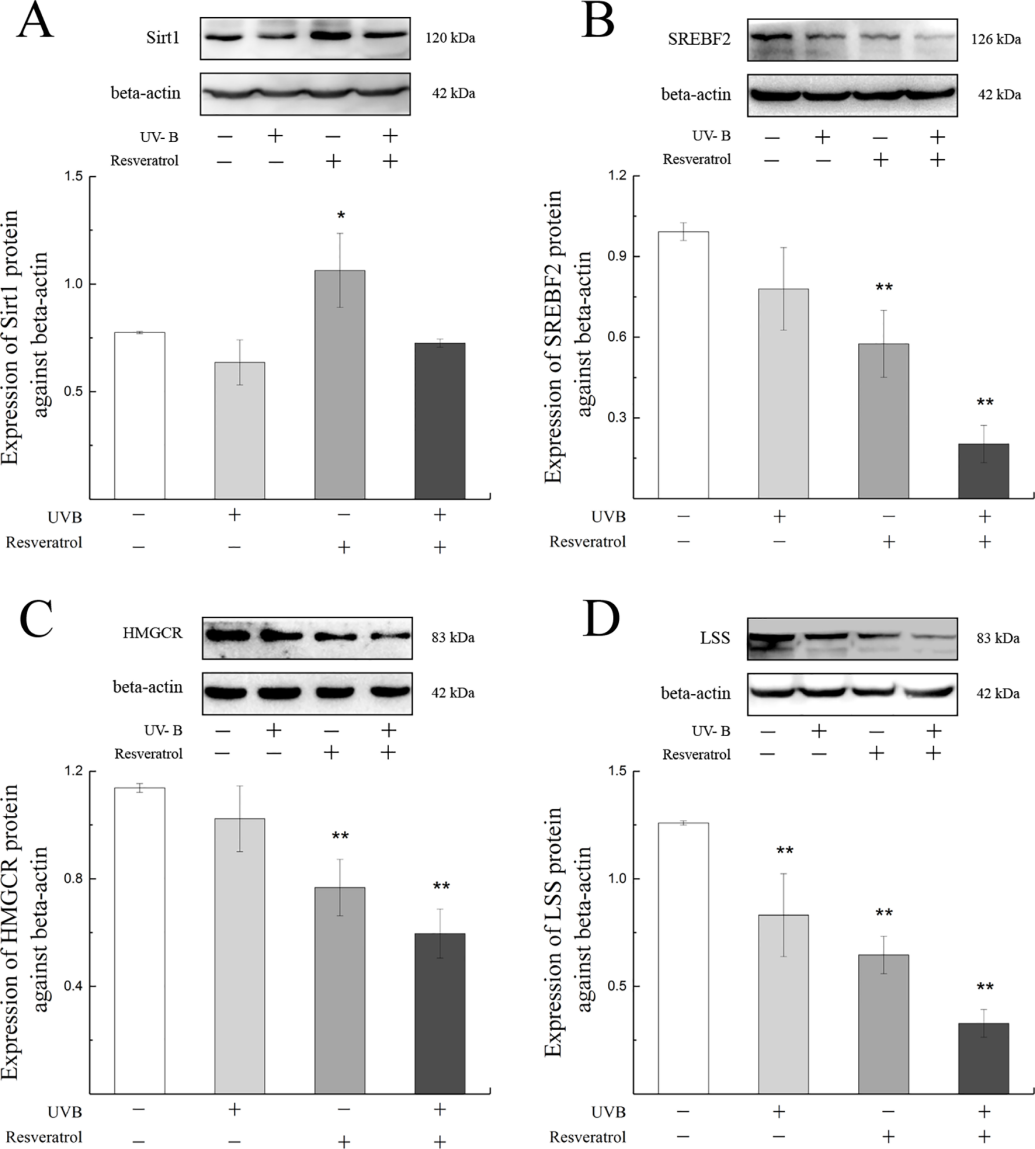
Sirt1, SREBF2, HMGCR, and LSS protein expression alterations in HLE cells pretreated with a Sirt1 agonist. The relative band densities of Sirt1, SREBF2, HMGCR, and LSS protein expressions were quantified relative to the expression of beta-actin, and the results were shown in **(A)** to **(D)** (n = 4). Data were expressed as the mean ± SD. *P < 0.05, **P < 0.01 compared with the control group.

As shown in **Figure 7**, the expression levels of Sirt1, SREBF2, HMGCR, and LSS proteins in the cells were decreased after exposure to UV-B compared with the levels found in the control group. To further explore whether the SREBF2, HMGCR, and LSS protein expressions were regulated by the Sirt1 expression level, we treated the HLE cells with a Sirt1 agonist. Resveratrol treatment was markedly down-regulated the expression levels of SREBF2, HMGCR, and LSS proteins compared with those found in the control group (*P* < 0.01), which contrasted with the results obtained with a significant increase in Sirt1 protein (*P* < 0.05). The protein expression levels of SREBF2, HMGCR, and LSS were significantly decreased in the resveratrol-pretreated group (pretreated with resveratrol and then exposed to UV-B) compared with the control group (*P* < 0.01).

## Discussion

UV-B radiation is a well-known risk factor for human eye injury (Andley et al., 2011), and lens epithelial cells are target cells of UV-B damage (Liou et al., 2015). UV-B radiation exposure could induce abnormal proliferation and differentiation of lens epithelial cells, resulting in cell apoptosis and necrosis and thereby the development of cataracts (Lan et al., 2016; Jeayeng et al., 2017). Crystallin is the most abundant protein in the lens, and previous studies have proposed that UV-B exposure caused the denaturation and aggregation of crystallin (Schafheimer and King, 2013). Lanosterol, which is synthesized by LSS, could significantly decrease the aggregation of crystallin (Zhao et al., 2015; Serebryany et al., 2016; Kang et al., 2018), and LSS inhibition could result in congenital cataracts (Chen and Liu, 2017). The process of UV-B radiation–induced cataracts is highly complex, and the molecular mechanisms, particularly during the early stages of this process, remain unknown. Therefore, we constructed *in vivo* and *in vitro* models to further explore the alterations in related UV-B-induced lens epithelial cell indices during the early stages of injury and the roles of LSS during the response to oxidative stress. Finally, we aimed to provide a mechanistic basis for further studies of UV-B damage and the development of strategies for the early prevention of cataracts.

H&E staining revealed that the degree of pathological injury increased with increases in the UV-B intensity, and the ROS content in the UV-B-exposed groups, showed a significantly dose-dependent increased compared with the control level. These data suggested that UV-B-induced oxidative stress in the lens epithelial cells and that most ROS were generated at the maximum UV-B dose, which might result in the apoptosis of lens epithelial cells. Under physiological conditions, crystallin could maintain the structure and physiological functions of the lens, whereas abnormal crystallin expression can induce cataracts (Makley et al., 2015). A previous study revealed that CRYAA could block the release of ROS from the mitochondria (Zhu et al., 2015) and inhibit apoptosis by enhancing phosphoinositide-3-kinase activity and inactivating the phosphatase and tensin homologue (Pasupuleti et al., 2010). In this study, we found that the CRYAA and CRYAB levels decreased dose-dependently after UV-B exposure, which suggested that the induction of ROS by UV-B could reduce the soluble crystallin content and that this process might also affect the apoptosis of lens epithelial cells. Therefore, we analyzed the apoptosis rate of lens epithelial cells and found a marked increase after UV-B exposure. These results indicated that UV-B exposure might induce oxidative stress in lens epithelial cells and thereby increased the apoptosis levels.

The content of MDA, the product of membrane lipid peroxidation, increased significantly after UV-B exposure, which was consistent with previous results (Ji et al., 2015). Under physiological conditions, ROS was eliminated by antioxidants or antioxidant enzymes in the body (Subedi et al., 2017). However, in this study, we found that the activities of SOD and GSH-Px and the content of GSH showed significant dose-dependent decreases with the increases in the UV-B intensity. These results might be due to the increase in ROS formation induced by UV-B, which resulted in inhibition of the activities of SOD and GSH-Px. GSH is an abundant nonprotein sulfhydryl compound in cells, and the resistance to ROS overproduction results in significant depletion of GSH. In addition, GSH-Px is the most important enzyme in GSH antioxidation (Shin et al., 2014; Terazawa et al., 2015). Decreases in GSH-Px activity and exhaustion of GSH could increase injuries to the antioxidant system, ultimately resulting in lens epithelial cell apoptosis. Previous studies proposed that Bax, Bcl-2, and caspase-3 were related to the apoptotic process (Gu, 2018). Bax is a member of the Bcl-2 family that promotes apoptosis and increases in the Bax and Bcl-2 ratio promote cell apoptosis (Du et al., 2017). Caspase-3 is the main executioner in the apoptotic caspase cascade, and the cleavage of caspase-3 is involved in the dismantling of cellular substrates during apoptosis (Hengartner, 2000). In this study, we found that increases in the UV-B intensity increased the expression levels of Bax and cleaved caspase-3 and the ratio of Bax to Bcl-2 at the mRNA and protein levels, and these findings were consistent with the results from the apoptosis analysis. These data indicated that UV-B exposure–induced apoptosis by initiating the caspase-3 pathway.

Lanosterol was previously reported to significantly decrease crystallin aggregation (Zhao et al., 2015; Serebryany et al., 2016; Kang et al., 2018), and strikingly, lanosterol is cyclized from squalene through a reaction catalyzed by LSS. Therefore, we analyzed the expression of LSS and found that the expression of LSS was increased during the early stage of the induction of oxidative stress induced by UV-B. In addition, LSS is an important rate-limiting enzyme in cholesterol synthesis pathway, and the expression of LSS might be regulated by a series of factors. Sirt1 was originally identified as an NAD-dependent histone deacetylase (Kalous et al., 2016). Recent studies have revealed that Sirt1 was involved in various cellular processes, such as the stress response, cell cycles, metabolism, apoptosis in response to cellular energy, and the redox status, *via* its deacetylase activity (Kalous et al., 2016) (Mvunta et al., 2017). Under physiological conditions, SREBF2 plays a canonical role in cholesterol homeostasis by transcriptionally regulating molecules involved in cholesterol biosynthesis. HMGCR, the rate-limiting enzyme in cholesterol synthesis, is an important target of SREBF2 (Li et al., 2017). Downstream of HMGCR, LSS catalyzes 2,3-oxide squalene cyclization to lanosterol in cholesterol synthesis (Fitzky et al., 2001; Goldstein et al., 2006). Therefore, we quantified the expression levels of Sirt1, SREBF2, and HMGCR in this study and found that Sirt1 expression in rat lens epithelial cells was decreased after UV-B exposure in a UV-B dose-dependent manner and that the expressions of SREBF2 and HMGCR increased with the increase in the UV-B intensity.

To further explore the function of increased LSS expression under UV-B-induced oxidative stress, we cultured HLE cells overexpressing LSS protein and observed alterations in apoptosis-related factors. Interestingly, after exposure to UV-B radiation, compared with the control group, the levels of apoptosis and ROS were significantly decreased in the LSS overexpression group, whereas the crystallin and LSS protein expression levels were increased. Moreover, the LSS overexpression group exhibited decreased MDA content, significantly increased activities of SOD and GSH-Px, and higher GSH content compared with the control group. Subsequently, the Bax and cleaved caspase-3 protein expression levels and the ratio of Bax to Bcl-2 were decreased in the LSS overexpression compared with the control group. In accordance with the lower apoptosis-related indicators in the overexpression LSS group as mentioned above, the cell viabilities at 24, 48, and 72 h were higher in the overexpression LSS group than those in the control group. Moreover, the overexpression of LSS can alleviate the aggregation of crystallin after UV-B exposure in HLE cells, and the increased soluble crystallin might be due to the disaggregation function of lanosterol. The increase in CRYAA might induce a decrease in the apoptosis rate which might be related to the caspase-3 pathway (Xu et al., 2015). Previous studies have also demonstrated that the addition of an inhibitor of oxidosqualene cyclase (OSC, LSS is a member of the OSC family) could induce the apoptosis of lens epithelial cell in intact rat lenses and rodents (Cenedella and Bierkamper, 1979; Pyrah et al., 2001), and it also could increase membrane structural order (and improve the membrane structure), which directly contributed to lens opacification (Cenedella et al., 2004). A mutation in the gene encoding LSS also lead to a congenital cataract in human (Chen and Liu, 2017). These results indicated that LSS was an essential enzyme in the maintenance of lens transparency, and the up-regulation of LSS might alleviate the damage of lens epithelial cells during the early stages of the induction of oxidative stress by UV-B.

To explore whether the decline in Sirt1 could regulate the expression of LSS, we analyzed the protein expressions of SREBF2, HMGCR, and LSS in HLE cells pretreated with a Sirt1 agonist. Consisting with our primary hypothesis, we found that the protein expressions of SREBF2, HMGCR, and LSS decreased with increases in Sirt1 protein expression compared with the control group. Similar results were recently found in previous studies with human fetal hepatocyte L02 cells (Hu et al., 2019). However, in contrast with the up-regulated of SREBF2, HMGCR, and LSS found in rat lens epithelial cells after UV-B exposure, HLE cells exposed to UV-B showed decreased expressions of SREBF2, HMGCR, and LSS proteins. A previous study demonstrated that the expression of Sirt1 in ARPE-19 cells increased and then decreased with increases in the UV-B dose (Chou et al., 2013). In addition, the expression of SREBF2 was up-regulated under moderate oxidative stress (Soundararajan et al., 2008). Therefore, we hypothesized that the expressions of SREBF2, HMGCR, and LSS protein in rats were up-regulated during the early stages of UV-B-induced oxidative stress due to the regulation of eye tissue as a whole (such as the antioxidases in the blood and aqueous humor). In contrast, HLE cells were poorly resistant to damage induced by UV-B, and after 24 h of exposure, the expression levels of SREBF2, HMGCR, and LSS were decreased. Nevertheless, we found that Sirt1 negatively regulated LSS and that the protein expressions of SREBF2, HMGCR, and LSS further decreased during UV-B exposure. Furthermore, the inhibition of Sirt1 transcription might provide negative feedback to SREBF2 mRNA and protein expression (Abe and Berk, 2013; Kim et al., 2013). In contrast, the increasing levels of oxidative stress could also activate SREBF2 transcription and translation (Chen et al., 2015). Therefore, we suspected that during the early apoptotic stages induced by UV-B, lens epithelial cells might attempt to increase their lanosterol content by increasing HMGCR and LSS expressions and inhibiting crystallin aggregation, and these effects would alleviate the resulting damage. This event might be a self-stress response of lens epithelial cells during the early stages of UV-B-induced oxidative stress. Because it served as a downstream gene, it was hypothesized that the up-regulation of LSS *via* the Sirt1 pathway during the early stage of UV-B-induced damage might be a resistance response oxidative stress.

In summary, our present study showed that UV-B exposure increased oxidative damage in lens epithelial cells and decreased the content of soluble alpha-crystallin, which resulted in cell apoptosis. The increased LSS expression observed during the injury process might inhibit the aggregation of crystallin at the early stages. LSS overexpression could alleviate apoptosis induced by UV-B, which also supported this conclusion. The increased LSS expression induced by UV-B might regulate *via* Sirt1. The addition of a Sirt1 agonist inhibited the expressions of SREBF2, HMGCR, and LSS proteins. However, the specific mechanisms remain unclear, and the detailed mechanisms of the Sirt1 pathway require further study. In our present study, it showed that LSS can maintain the physiological structure of crystallin and reduce the apoptosis rate during the induction of oxidative stress by UV-B, and thus, LSS might be an important target in the prevention of cataracts.

## Conclusion

UV-B exposure induced oxidative damage, resulting in crystallin denaturation and lens epithelial cell apoptosis. LSS could play a protective role in the early stages of this process.

## Data Availability

The raw data supporting the conclusions of this manuscript will be made available by the authors, without undue reservation, to any qualified researcher.

## Ethics Statement

This study was carried out in accordance with the recommendations of “National Institutes of Health guidelines, China Medical University Committee on Ethics in the Care and Use of Laboratory Animals” (specific pathogen-free [SPF] grade; Permit Number: SCXK-2015-0001). The protocol was approved by the “China Medical University Committee.” The experiments were performed in strict accordance with the Guide for the Care and Use of Laboratory Animals of the National Institutes of Health. All surgeries were performed under anesthesia, and all efforts were made to minimize suffering.

## Author Contributions

YL and TY contributed to the conception of the study. HH and TY conducted most of the experiments, analyzed the data, and wrote this manuscript. LH, ML and ZZ contributed to the part of experiment preparation. RC, NW, and DY helped perform the data analyses. All authors approved the final version of this manuscript.

## Funding

Support for this study was provided by the National Natural Science Foundation of China [grant numbers NSFC 81673133].

## Conflict of Interest Statement

The authors declare that this study was conducted in the absence of any commercial or financial relationships that could be construed as a potential conflict of interest.
