# Supplementary material for: Protective Effects of Lanosterol Synthase Up-Regulation in UV-B-Induced Oxidative Stress
Source: Front Pharmacol. 2019 Aug 29;10:947. doi: 10.3389/fphar.2019.00947 (PMC6726740; doi:10.3389/fphar.2019.00947)

**Supplemental Material**

**Supplemental Material 1_Supplemental Material Figure 1:**


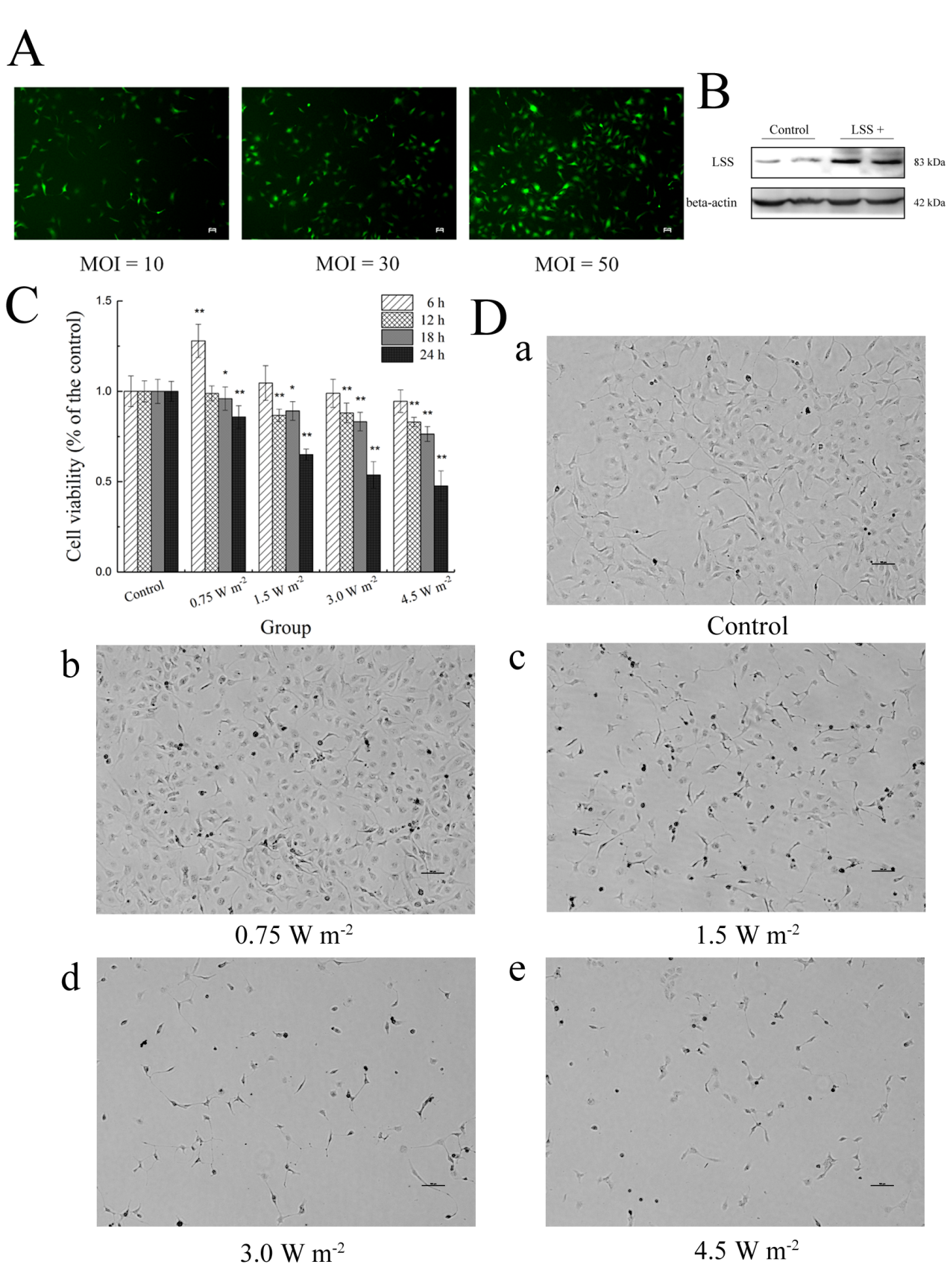


**Supplemental Material Figure 1: Determination of transfection MOI and exposure irradiance in HLE cells.** Fluorescence-positive HLE cells in different MOIs are shown in the S Fig. 1A , scale bar indicates 50 μm. Western blot protein bands of control and LSS overexpression group were shown in S Fig. 1B. The cell viabilities in different UV-B exposure group for 120s were shown in S Fig. 1C (n=6). Data were shown as the mean ± SD. **P*<0.05, ***P*<0.01 compared with the control group. Morphological changes of HLE cells after exposed to different UV-B irradiances 24 h later are shown in S Fig. 1D a -e (40 ×).

**Supplemental Material 2_ Supplemental Material Figure 2:**

**
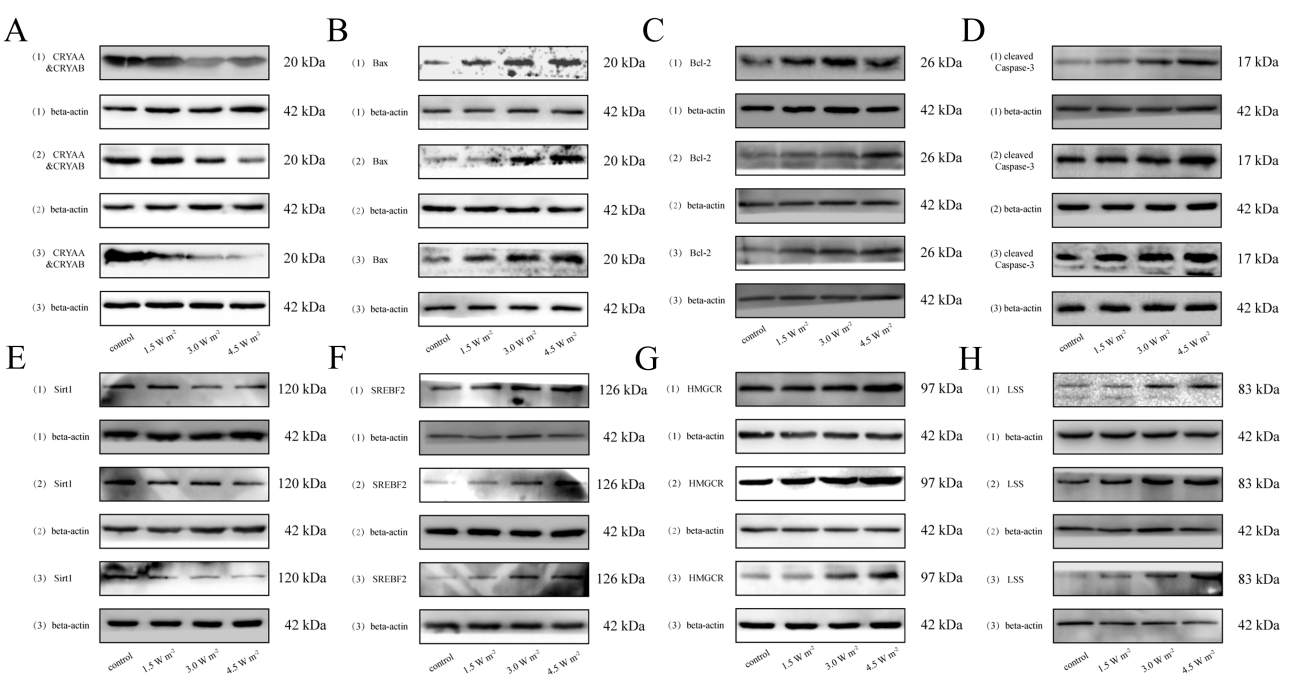
**

**Supplemental Material Figure 2:** **Western blot protein bands of rat lens used for analysis in the study.** Beta-actin was used as the loading control. (A) Protein expression of CRYAA and CRYAB. (B) Protein expression of Bax. (C) Protein expression of Bcl-2. (D) Protein expression of cleaved Caspase-3. (E) Protein expression of Sirt1. (F) Protein expression of SREBF2. (G) Protein expression of HMGCR. (H) Protein expression of LSS. (* The beta-actin in D (2) is the same with the beta-actin in E (3))

**Supplemental Material 3_ Supplemental Material Figure 3**

**
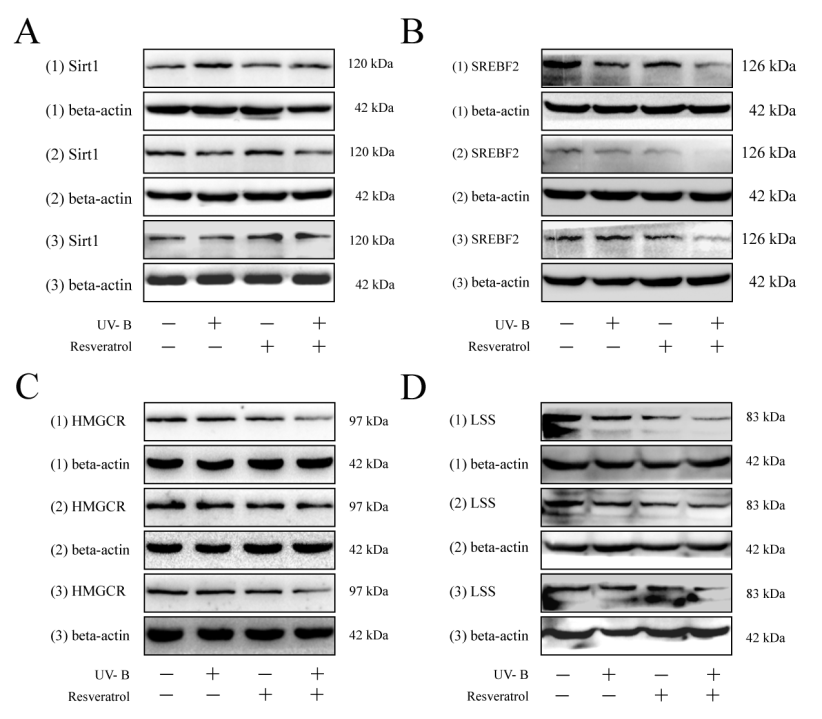
**

**Supplemental Material Figure 3: Western blot protein bands of HLE cells used for analysis in the study.** Beta-actin was used as the loading control. (A) Protein expression of Sirt1. (B) Protein expression of SREBF2. (C) Protein expression of HMGCR. (D) Protein expression of LSS.

**Supplemental Material 4_Cell lines SRA01/04[HLE] STR profile report.**


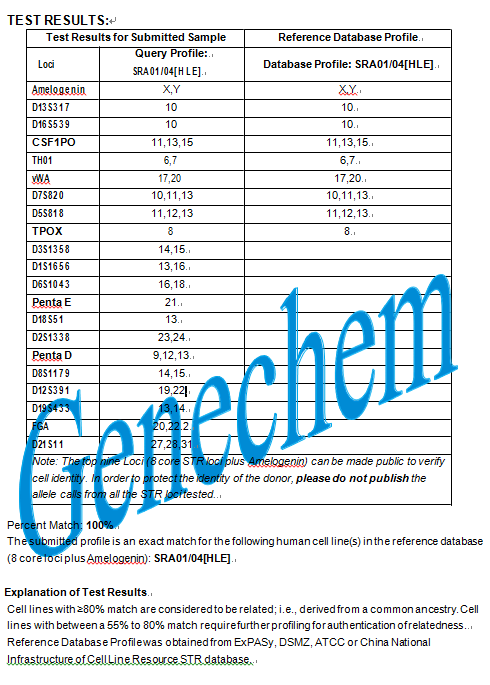


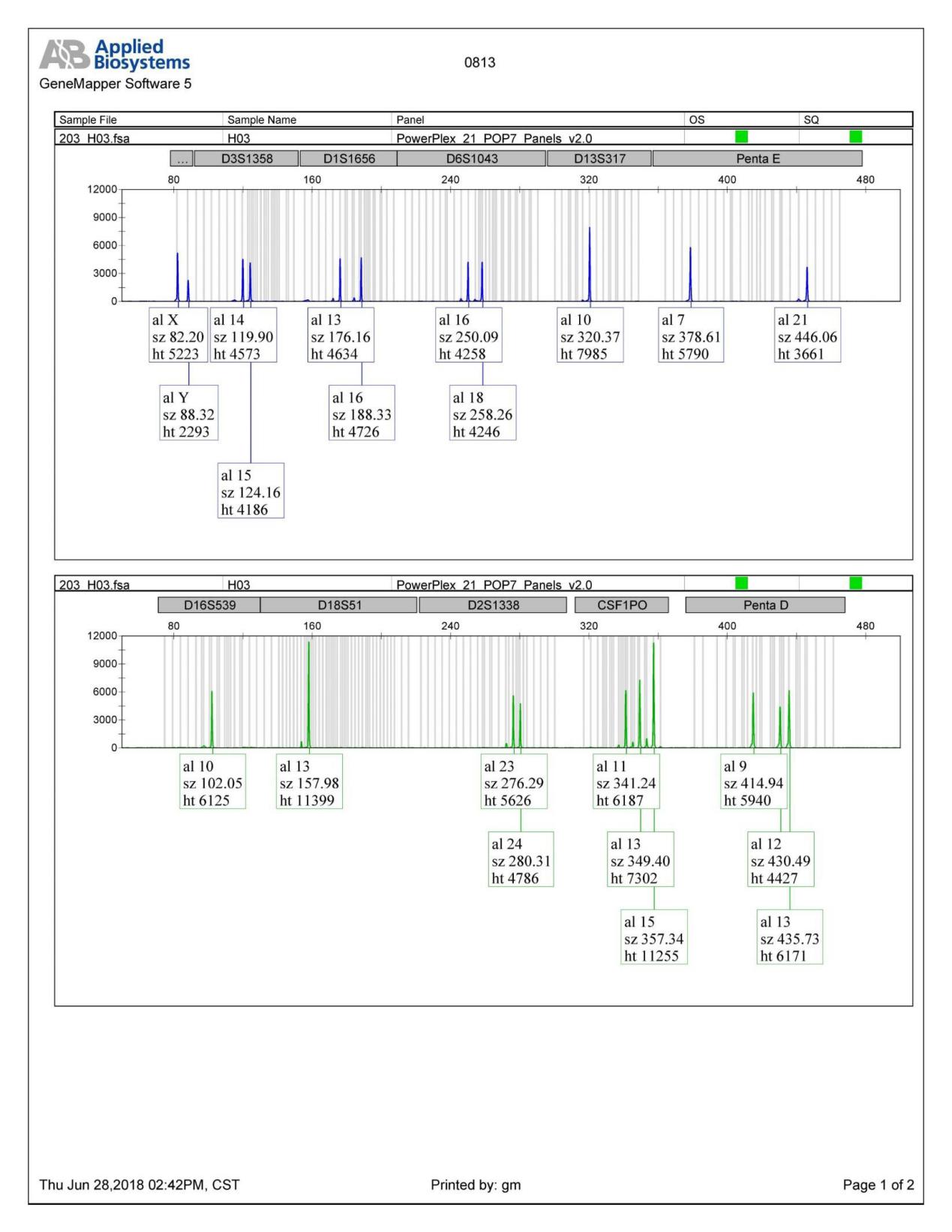

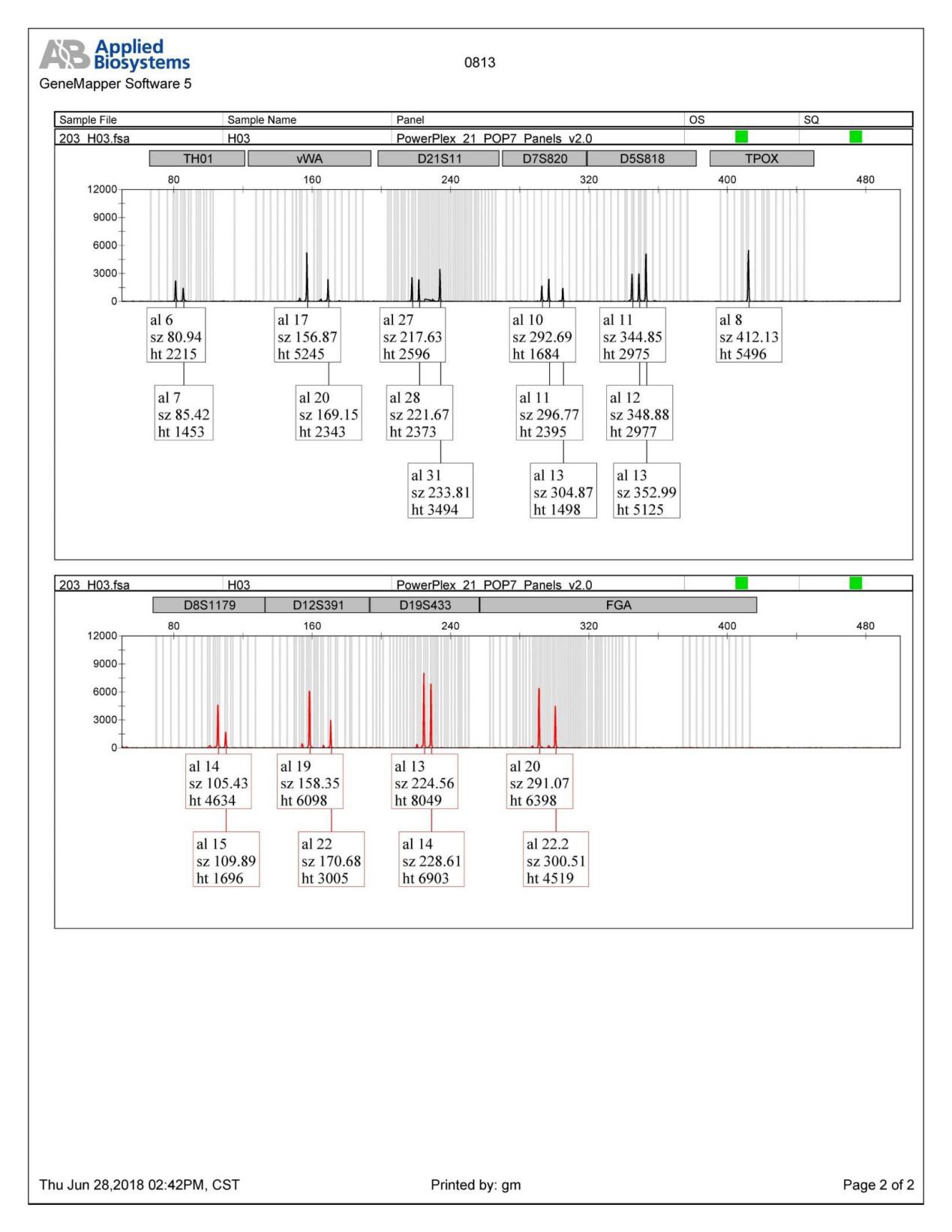

Supplement: Supplementary file 1 [file Table_1.docx]
